# Change in hospital antibiotic use and acquisition of multidrug-resistant gram-negative organisms after the onset of coronavirus disease 2019

**DOI:** 10.1017/ice.2020.1360

**Published:** 2020-12-10

**Authors:** Jacqueline T. Bork, Surbhi Leekha, Kimberly Claeys, Hyunuk Seung, Megan Tripoli, Anthony Amoroso, Emily L. Heil

**Affiliations:** 1 Division of Infectious Diseases, Department of Medicine, University of Maryland School of Medicine, Baltimore, Maryland; 2 University of Maryland School of Epidemiology and Public Health, Baltimore, Maryland; 3 University of Maryland School of Pharmacy, Baltimore, Maryland

## Abstract

Interrupted time series segmented regression was conducted to trend antibiotic use and multidrug-resistant gram-negative (MDRGN) acquisition relative to COVID-19 in an academic hospital. Total antibiotic use and antibiotic use related to pneumonia was higher in the period after the onset of COVID-19 compared to the similar calendar period in 2019. Furthermore, MDRGN acquisition increased 3% for every increase in positive COVID-19 tests per week.

The coronavirus disease 2019 (COVID-19) pandemic has created innumerable challenges for healthcare systems. In the pandemic’s early phases, controlling COVID-19 transmission and managing patients with COVID-19 effectively was emphasized.^1^ The consequences of this shift to COVID-19–focused care on other aspects of infection control and antimicrobial stewardship have yet to be fully explored.

Reported antibiotic use in COVID-19 patients is high (71%–100%), although bacterial coinfection rates are lower (5%–39%), likely reflecting perceived high risk for secondary infection due to critical illness.^2-4^ However, the impact of COVID-19 on antimicrobial stewardship in the broader hospital population needs further investigation.^5,6^ Herein, we describe trends in overall and pneumonia antibiotic use and the incidence of multidrug-resistant gram-negative organisms (MDRGNs) at an academic medical center relative to the onset of the COVID-19 pandemic.

## Methods

We conducted this study at the University of Maryland Medical Center, a 757-bed acute-care hospital, and referral center for COVID-19 in Maryland. This hospital has 4 designated COVID-19 units (2 are ICU units). An infectious diseases consultation service evaluated all patients with confirmed or suspected COVID-19. The first COVID-19 patient was admitted on March 14, 2020. Maryland state lockdown restrictions spanned March 30, 2020 (week 14), to April 14, 2020 (week 20).

We examined the 24-week period from January 5 to June 27 in 2020 for COVID-19–related trends, using the same period in 2019 as a control. COVID-19 cases began to increase in week 14 of 2020; thus, we considered weeks 2–13 as the pre–pandemic-onset period and weeks 14–25 as the post–pandemic-onset period. Weekly antibiotic days of therapy per 1,000 patient days (DOT per 1,000 PD) were calculated using the hospital’s antimicrobial stewardship database, which includes daily dispensed antimicrobials, associated indication, and hospital census for the following categories: (1) total antibiotics, (2) pneumonia antibiotics and (3) early pneumonia antibiotics (<7 days from hospital admission). Early pneumonia data captured suspected community-onset bacterial coinfections (as opposed to hospital-onset infections). To account for the decrease in patient days in 2020 driving changes in antibiotic DOT per 1,000 PD, we also separately analyzed the proportions of antibiotic DOTs for pneumonia and early pneumonia DOTs in the 2019 and 2020 post–pandemic-onset periods as well as the monthly proportions of COVID-19 patients who received antibiotics for pneumonia monthly in 2020.

We measured MDRGN incidence as the number of clinical cultures (ie, first culture per patient and cultures >48 h after admission) per 10,000 patient days (MDRGN per 10,000 PD) growing Enterobacterales, *Pseudomonas aeruginosa*, or *Acinetobacter baumannii* that were nonsusceptible to ≥2 of the following antimicrobial agents: piperacillin/tazobactam, cefepime, or carbapenem. We calculated hospital-wide MDRGN incidence and MDRGN incidence among COVID-19–specific patients (using COVID-19 patient days).

### Statistical analysis

An interrupted time series was conducted using segmented regression, a generalized linear model (GLM) with negative binomial or Poisson distribution. Models allowed for variation in the intercept (β0), the pre-onset slope (β1), the post-onset level change (β2), the post-onset slope change (β3), the pre-onset level difference between 2020 and 2019 (β4), the pre-onset slope difference between 2020 and 2019 (β5), the post-onset level difference between 2020 and 2019 (β6), change in slope difference pre-onset to post-onset between 2020 and 2019 (β7), and the COVID-19 burden as follows:
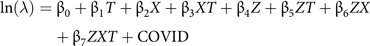



where *λ* = rates, *T* = time (week), *X* = study phase (COVID-19), *XT* = time after COVID-19 onset, *Z* = year 2020 or year 2019 (control), *ZT* = time for 2020 and 0 for control, *ZX* = COVID-19 onset for 2020 and 0 for control, *ZXT* = time after COVID-19 onset for 2020 and 0 for control, and COVID = positive tests per week. Both immediate (level) and gradual (slope) changes post COVID-19 onset were estimated, along with “difference-in-difference” from the control period (see Supplementary Material online for details). Categorical variables were compared using the χ^2^ test. A *P* value <.05 was considered statistically significant. Analyses were performed using SAS version 9.4 software (SAS Institute, Cary, NC) and R Core Team (2020).

This study was deemed nonhuman subject research by the University of Maryland Institutional Review Board.

## Results

**Fig. 1.**
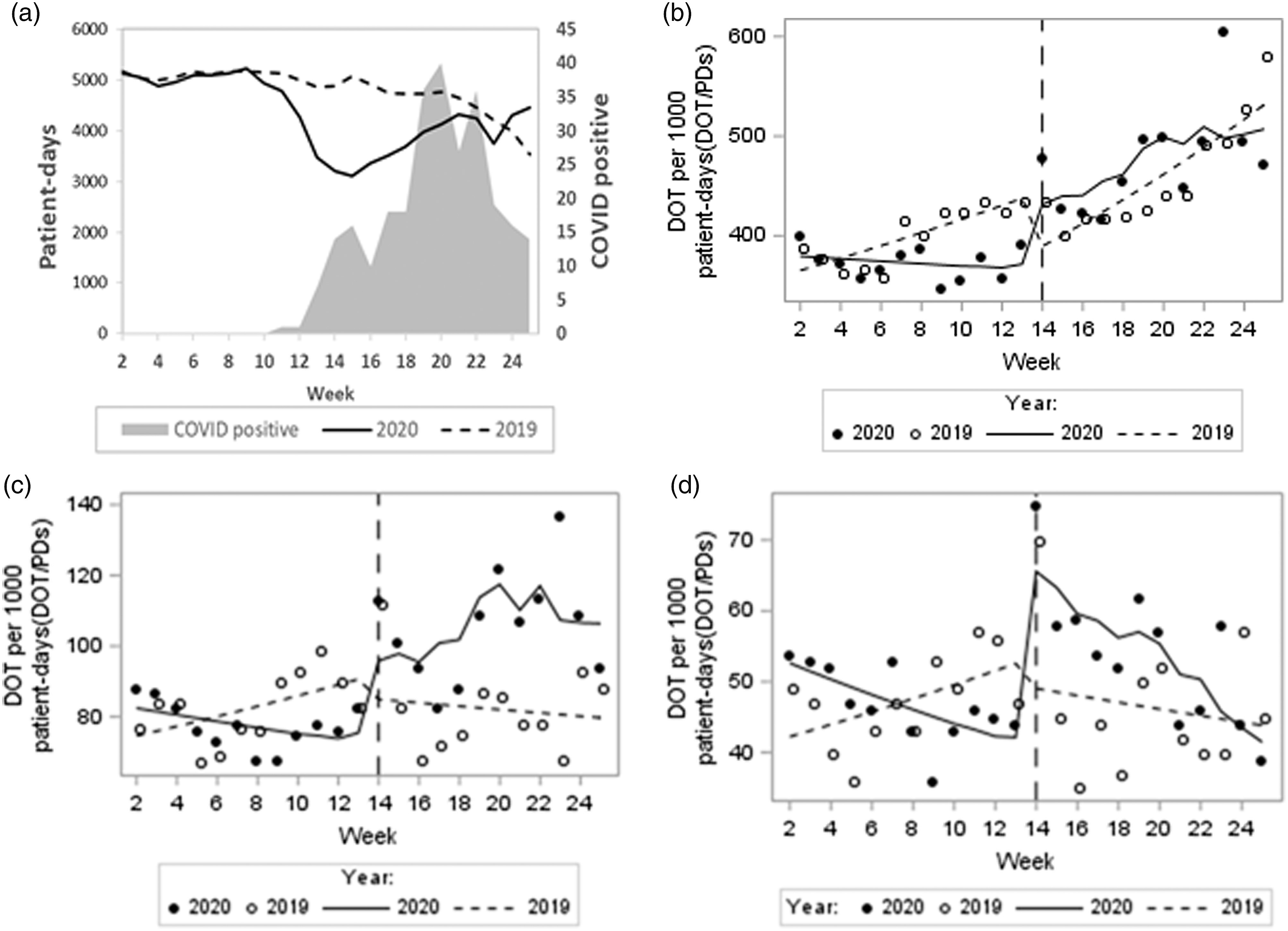
(a) Patient days relative to COVID-19–positive inpatients. (b) Antibiotic days of therapy per 1,000 patient days (DOT/PDs) over time. (c) Pneumonia antimicrobial days of therapy per 1,000 patient days (DOT/PDs) over time. (d) Early pneumonia (within 7 days of admission) antimicrobial days of therapy per 1,000 patient days (DOT/PDs) over time. Note. the observed values and predicted means (trend) are presented as circles (filled or not filled) and lines (solid or short dash), respectively.

There were 400 antibiotic DOT per 1,000 PD in 2019 during the pre-onset period and 453 antibiotic DOT per 1,000 PD in 2019 during the post-onset period. There were 373 antibiotic DOT per 1,000 PD in 2020 during the pre-onset period and 479 antibiotic DOT per 1,000 PD in 2020 during the post-onset period, respectively. In 2020, patient days reached a nadir in week 15 and COVID-19 cases reached a peak at week 20 (Fig. 1a). In 2020, COVID-19 patient days were 6% of the total patient days for the overall study period. For total antibiotics use, the 2020 post- onset period level (DOT per 1,000 PD) increased by 13% (*P* = .06), with a difference-in-difference of levels between 2019 and 2020 of +30% (*P* = .0003). The rate of DOT per 1,000 PD increased by 1.8% per week during the post-onset period in 2020 (*P* = .02), without a significant change in the difference-in-difference of slopes between 2019 and 2020 (+0.61%; *P* = .50) (Fig. 1b).

For pneumonia indication, the 2020 post-onset period level (DOT per 1,000 PD) increased by 21% (*P* = .057), with a difference-in-difference of levels between 2019 and 2020 of +28% (*P* = .07). The rate of DOT per 1,000 PD increased by 2.1% per week during the post-onset period (*P* = .10), with a difference-in-difference of slopes between 2019 and 2020 of +5% (*P* = .02) (Fig. 1c). For early pneumonia indication, the 2020 post-onset period level (DOT per 1,000 PD) increased by 59% (*P* = .0003), with a difference-in-difference between 2019 and 2020 of +68% (*P* = .0003). The rate of DOT per 1,000 PD decreased by 2% per week during the 2020 post-onset period (*P* = .30) without a significant difference-in-difference of slopes between 2019 and 2020 (+1%; *P* = .70) (Fig. 1d). The proportions of pneumonia and early pneumonia antibiotic days in 2020 versus 2019 during the post-onset period were 22% vs 18% (*P* < .0001) and 11% vs 10% (*P* = .0043), respectively. Among 293 COVID-19 patients, the proportions receiving antibiotics for pneumonia in March, April, May, and June were 84% (16 of 19), 69% (42 of 61), 42% (34 of 82), and 30% (12 of 40), respectively (*P* < .0001).

**Fig. 2.**
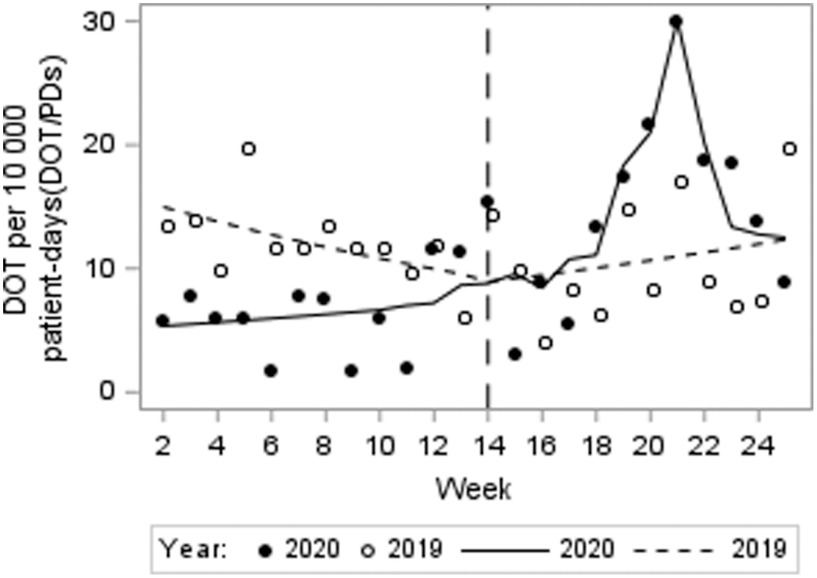
Incidence of multidrug-resistant gram negatives per 10,000 patient days (DOT/PDs) over time. Note. The observed values and predicted means (trend) are presented as circles (filled or not filled) and lines (solid or short dash), respectively.

For MDRGN acquisition, neither the immediate 2020 post-onset period level change (−18%; *p* = .50) nor the trend (0%; *P* = .90) was significant. Similarly, differences were not significant relative to the same period in 2019: the difference-in-difference of levels between 2019 and 2020 was −10% (*P*= .80) and the difference in slope change was −6% (*P* = .30) (Fig. 2). However, in 2020, for every positive COVID-19 test per week, there was a 3% increase in MDRGNs per 10,000 PD (*P =* .002). In the 2020 post-onset period, the MDRGN per 10,000 PD for COVID-19 patients was 68 compared to 17 hospital-wide, and COVID-19 patients were 24% of all MDRGN cases.

## Discussion

Antibiotic utilization in an academic center after the onset of the COVID-19 pandemic increased compared to a similar control period in 2019. After the onset of COVID-19, increases in antibiotic DOT per 1,000 PD were observed for indications of pneumonia (gradual) and early pneumonia (immediate); these trends were not detected during comparable 2019 periods. Antibiotic days related to pneumonia as a proportion of total antibiotic days was also higher in the 2020 post-onset period than in 2019.

At our institution early in the pandemic, of antibiotics targeting concomitant bacterial infections in patients with confirmed or suspected COVID-19 pneumonia were frequently administered. Similar high levels of antibiotic prescribing for pneumonia in academic centers during the pandemic have been reported.^6,7^ Antibiotic overuse may have been secondary to prolonged turnaround time for COVID-19 test results, preventing timely differentiation of COVID-19 from bacterial pneumonia. Also, limited knowledge of COVID-19 clinical manifestations and observed rates of bacterial superinfection compared to other respiratory viral illnesses such as influenza may have played a role in prescribing.^7^

To evaluate the downstream effects of antibiotic use, we evaluated MDRGN acquisition given that antibiotic exposure is a driver of MDRGN colonization and infection.^8^ Although MDRGN incidence did not differ significantly during the 2020 post-onset period compared to the same period in 2019, it increased among COVID-19 patients. This increase is likely multifactorial in etiology (eg, altered infection control practices, critical illness, and prolonged hospital stay of COVID-19 patients). However, high antibiotic utilization may have contributed to the increase in MDRGNs among COVID-19 patients.^9^

The strengths of our study include the use of a 2019 control period and a difference-in-difference approach. The limitations of this study include the single-center experience and the lack of adjustment for seasonality and unmeasured confounders (eg, infection control and antimicrobial stewardship practices). As the COVID-19 pandemic continues, antibiotic stewardship efforts are essential to preventing unnecessary antibiotic use and the further emergence of antibiotic resistance.
